# Impact of an education intervention on knowledge of high school students concerning substance use in Kurdistan Region-Iraq: A quasi-experimental study

**DOI:** 10.1371/journal.pone.0206063

**Published:** 2018-10-31

**Authors:** Nazar Mahmood, Samir Othman, Namir Al-Tawil, Tariq Al-Hadithi

**Affiliations:** Department of Community Medicine, College of Medicine, Hawler Medical University, Erbil, Iraq; University of Catania, ITALY

## Abstract

**Background and aims:**

Substance use among adolescents especially smoking, is becoming a public health problem in the Kurdistan region of Iraq. School-based health education is an appropriate approach for improving students’ knowledge regarding substance use in an attempt to prevent or reduce such problem The purpose of the study was to examine the effect of an educational intervention for high school students to improve their knowledge for substance use and its negative consequences, which will, in turn, motivate students to take protective measures against substance use.

**Methods:**

This quasi-experimental (one group; pre, and post-test) design was carried out in Erbil city from January 2017 to June 2017. A random sampling technique was employed to collect a sample of 280 students amongst four high schools in Erbil city which is the capital of Kurdistan Region-Iraq. A self- administered questionnaire on knowledge assessment regarding substance use was developed and validated by the experts regarding the relevance of the items. A structured teaching program for imparting knowledge on various aspects of substance use was developed based on extensive review of literature and experts’ opinion. The intervention program consisted of a series of 4 education modules. These modules were mainly taught by “Rabers” over a period of four weeks (one session per week). SPSS version 21 was used for data entry and analysis. Data was analyzed through descriptive and inferential statistics (McNemar tests, paired *t*-test, and Chi-square test).

**Results:**

Out of 280 students, a total of 270 students completed a pre and post-intervention survey. Of the 270 students, 124 (45.9%) were males and 146 (54.1%) were females. The mean age ± SD of the participants was 16.59 ± 0.784 years, ranging from 15–18 years. The study reveals a statistically significant improvement in the mean score of knowledge of students following the implementation of a health education program from 15.959 ± 3.25 to 20.633 ± 3.26 (*p* < 0.001). Moreover, no one of the students remained with poor knowledge, and relatively more than half (50.2%) of the students have upgraded to good knowledge level.

**Conclusion:**

Implementing a health education program about substance use on high school students in Erbil city had improved the knowledge of students about this topic.

## Introduction

Substance use (alcohol, tobacco, and illicit drugs) are the major concerns of today’s world [1]. It is impairing human will and vanishing his mind in such a way that pushes them to commit crimes [2]. Therefore, substance use, of which every developed and developing community suffer, is considered one of the most complicated problems that face the community which is not less dangerous than terror [3].

Iraq is one of poor-income, conflict-affected countries that have a series of challenges in mental health, because of its continuous exposure to large-scale affective events like successive wars (from 1980 to present), economic embargo, organised continuous violence and terrorism [4]. These unsafe conditions mirrored negatively on the psychosocial status of the Iraqi community, mainly the children and youth who were hardly influenced by these events, by suffering diseases, psychological shock and death [5]. In addition, the geographic location of Iraq is another factor that makes Iraq vulnerable to substance use, this is because of the long and porous border between Iraq and Iran, as Iran faces an increasing and very serious problem in substance use [6]. According to the more recent report, the prevalence of lifetime uses of (Alcohol, Licit or Illicit Drugs) in Iraq (Including Kurdistan region) was approximately 10.3% [7].

Substance use extends through different age levels, but it seems to be more common among adolescents which are usually in high school and college [8], as adolescence is a critical life-course period during which patterns of health behaviour are formed, before tracking into adulthood, and they experience physical, mental, and social interactional changes during this period [9]. Moreover, adolescence is characterised by an increased adventurous tendencies and peer influences. As a result, adolescents are vulnerable to new things including the use of the substances [10].

In Iraq (including Kurdistan region), studies have shown that substance use by young people is on the rise. For example, according to the report of the Iraqi Community Epidemiology Work Group, there has been an increase in the use of alcohol, prescription drugs, and illicit drugs in Iraq, especially among the young people [11]. The most recent study among high school students in Erbil city has found that 41.7% of students are smokers, and this deems the alarming point, as smoking considered a gate for using other illicit substances, this may, in turn, progress to the use of other drugs [12].

Several factors influence whether an adolescent tries substances, and knowledge is one of the factors that influence students’ decision to use substances [9], with inadequate knowledge about the substance use and its consequences, a student will be less likely to make a fact-based and informed decision [13,14]. Studies have shown that providing youth with accurate information about negative consequences of substance use will encourage abstinence from substance use [15–17]. School-based health education is an appropriate approach for improving students’ knowledge regarding substance use [18]. Hence, providing substance use education in schools can encourage students to make positive decisions on their future lives, this, in turn, prevents or reduces the use of substances among this segment of population. This approach is influential in the field because as far as we know, no previous research has investigated an educational program about substance use for adolescents in the Kurdistan region of Iraq, and it will attempt to fill the gap in the literature, especially, Kurdistan region. Thus, the purpose of this study was to examine the effect of an educational intervention for high school students to improve their knowledge for substance use and its negative consequences, which will, in turn, motivate students to take protective measures against substance use.

## Methods

### Design and sample

This quasi-experimental (one group; pre, and post-test) design was conducted from January 2017 to June 2017 amongst high school students in Erbil city which is the capital of Kurdistan Region-Iraq. According to the data obtained from Erbil Directorate of Education, the total number of high schools was 78 in Erbil city during the academic year 2016–2017. Out of the 78 schools, only four schools (two for males and two for females) had been selected by simple random sampling technique using Microsoft Excel program. Students of grade 10 and 11 from these four schools were the target population for the educational program. A single class from grades 10, and 11 was randomly selected from each school, and then in the selected classes, 35 students were chosen randomly, knowing that the total number of students in each class is more than 35. As a result, 280 students in the age range of 15–18 were recruited to participate in the study. It is worthwhile to mention that high schools in Kurdistan Region-Iraq are consisting of three grades (10, 11, and 12). In this study, grade 12 was excluded because the students in this grade finish their classes and take an early break to prepare for the final exam; hence it is difficult to follow them up.

### Instrumentation

A self-administered questionnaire was developed by the researchers, based on the extensive review of literature, as a tool for data collection. The questionnaire consisted of two sections (S1 File). The first section related to socio-demographic items such as age, gender, educational level of parents, occupational status of father, and monthly family income which was categorised into four groups namely, more than enough, enough, barely enough, and less than enough. The second section was designed to assess students’ knowledge of substance use through 30 knowledge-testing items. Knowledge-testing questions were multiple choice questions. The majority of these questions were taken from both education program and booklet. A score of 1 was given to the correct answers and 0 to wrong answers. Scores of knowledge ranged from 0 to 30 with a higher score representing better knowledge about substance use. The level of knowledge was described as good if they scored 20–30, moderate if 10–19, and poor if 0–9. Prior to the actual study, a pilot test was done to determine the reliability of the questionnaire. Reliability was measured using the Cronbach’s alpha method with a value of 0.78. This result demonstrated that the questionnaire was internally consistent. The content validity the questionnaire was confirmed by a panel of four experts; two consultant psychiatrists (substance use), and two community medicine specialists. Based on the experts’ comments, only minor modifications to the wording of the content were required.

### The educational program

A structured teaching program for imparting knowledge on various aspects of substance use especially prevention was developed by the authors. The content of the educational curriculum was designed based on extensive review of literature and experts opinion. The intervention consisted of a series of 4 education modules. Module content was created and edited by the researchers; the first module included an introduction to substance use and historical background, some definitions, side effects and negative consequences, and causes and risk factors. The second and third modules identified the types of substance use and the final module described prevention and control of substance use. Before the curriculum is finalised, it has been sent to three experts. The three experts have read and edited the content of the curriculum to assure that the program fits local teaching and learning styles.

### Procedure

After ensuring informed consent from the students’ parents, the students in the four schools were given the pre-test questionnaire one week before administration of the educational program. The questionnaire was administered in the classrooms by schools’ social workers "Rabers" in the presence of the primary author. Each student was given a serial number to be followed in the second assessment (post-test). After administration of the questionnaire, four ‘45 minute-sessions’ were designed using lectures, group discussion, and booklets. These sessions were mainly taught by “Rabers” during four weeks (one session per week). These “Rabars” were trained properly by the primary author on the content of the educational program. As a reminder, each participant student was provided with a copy of the health education booklet prepared and designed by the primary author and reviewed by other authors, entitled "Know the Facts about Substance Use" (In the Kurdish language). The content of the booklet was similar to that of the educational program and it summarized the most important points in the program. One month later, students were asked to complete the same questionnaire a second time (post-test).

### Data analysis

The data were coded and entered into the Statistical Package for the Social Sciences (SPSS) software, version 21, and analyzed. McNemar test was conducted as univariate analysis to compare pre- to post-intervention changes in the percentage of students’ knowledge of each levels (low, moderate, and high). A paired *t*-test compared pre to post-intervention scores. Chi-square test was used to examine group differences. A P value of ≤0.05 was considered as the level of significance for all analyses.

## Results

Out of 280 students, a total of 270 students (96.4% response rate) completed a pre and post-intervention survey: 124 (45.9%) males and 146 (54.1%) females. The mean age ± SD of the participants was 16.59 ± 0.784 years, ranging from 15–18 years.

The result reveals a statistically significant improvement in mean knowledge score of students following the implementation of a health education program from 15.959 ± 3.25 to 20.633 ± 3.26 (p < 0.001) (Table 1).

**Table 1:** Comparison of mean of knowledge scores before and after educational program (N = 270).

Knowledge scores	Mean	Mean difference	SD	df	t-test	P value
Pre-test	15.959	4.6741	3.8297	269	-20.055	<0.001
Post-test	20.633					

There is no significant association between the gain in knowledge scores (post minus pre-test scores) and certain sociodemographic variables such as gender, age, education of parents, and occupational status of fathers, house ownership, and monthly family income (Table 2).

**Table 2:** Association between sociodemographic variables with the difference in knowledge scores (post minus pre-test scores).

Variables	N (270)	Post-test benefit scores *		*P*
		Low benefit	High benefit	
		No. (%)	No. (%)	
**Gender**				
Male	124	64 (51.6)	60 (48.4)	0.097
Female	146	90 (61.6)	56 (38.4)	
**Age (Years)**				
15-16	135	77 (57.0)	58 (43.0)	1.000
17-18	135	77 (57.0)	58 (43.0)	
**Educational level of father**				
Illiterate	29	16 (55.2)	13 (44.8)	0.377
Read and write	40	21 (52.5)	19 (47.5)	
primary school	43	24 (55.8)	19 (44.2)	
Intermediate school	49	30 (61.2)	19 (38.8)	
Secondary school	28	21 (75.0)	7 (25.0)	
Institute/College	81	42 (51.9)	39 (48.1)	
**Educational level of mother**				
Illiterate	84	47 (56.0)	37 (44.0)	0.130
Read and write	38	21 (55.3)	17 (44.7)	
Primary school	56	31 (55.4)	25 (44.6)	
Intermediate school	40	27 (67.5)	13 (32.5)	
Secondary school	16	9 (56.3)	7 (43.7)	
Institute/College	36	19 (52.8)	17 (47.2)	
**Occupational status of father**				
Employed	235	131 (55.7)	104 (44.3)	0.298
Un employed	13	7 (53.8)	6 (46.2)	
Retired	22	16 (72.7)	6 (27.3)	
**House ownership**				
Owned	244	142 (58.2)	102 (41.8)	0.238
Rented	26	12 (46.2)	14 (53.8)	
**Monthly family income**				
More than enough	16	6 (37.5)	10 (62.5)	0.339
Enough	118	72 (61.0)	46 (39.0)	
Barely enough	124	69 (55.6)	55 (44.4)	
Not enough	12	7 (58.3)	5 (41.7)	
**Total**	**270**	**154 (57.0)**	**116 (43.0)**	

* The median of benefit scores was 14.

-Low benefit: ≤ 14

-High benefit: > 14

Pre-test knowledge shows that 82.6% of the students had a moderate knowledge and about 4.1% of the students had poor knowledge whereas only 13.3% had good knowledge. An improvement in students’ knowledge has been shown immediately after program implementation, as significant differences were found between the levels of knowledge after educational program compared to its level before (Table 3). It is clearly shown that 63.6% of students with poor knowledge have upgraded to moderate knowledge and 36.4% upgraded to good knowledge. Regarding the students with moderate knowledge, half (50.2%) of them have upgraded to good knowledge and 49.8% remained as having moderate knowledge.

**Table 3:** Level of knowledge of students (N = 270) before and after educational program.

Pre-test level of knowledge	Post-test level of knowledge				Total
	Moderate		Good		
	No.	(%)	No.	(%)	
Poor	7	(63.6)	4	(36.4)	11
Moderate	111	(49.8)	112	(50.2)	223
Good	0	(0.0)	36	(100.0)	36

## Discussion

Having knowledge and information is the first key and necessary element in developing healthy behavior [14]. There are some evidences about the effectiveness of the school-based programme in raising knowledge of the students regarding the harms of substance use, which will, in turn, contributes to preventing or reducing it among students [9,14,19]. Numerous studies cited inadequate knowledge of students regarding substance use as a contributing factor for abusing it [9,13,20]. Students can construct their knowledge about substance use from different sources such as peers, media, family, and school [9]. However, the most appropriate source of information is a school-based programme [1], that is adopted by many countries, except Iraq, and more specifically the Kurdistan region of Iraq. So that, this study was conducted with the aim of assessing the effectiveness of a health education programme to improve students’ knowledge regarding substance use.

Prior to implementation of the education programme, the majority of high school students were at a moderate level and this level of knowledge was consistent with that reported by Geramian et al [13] and Rockville [21]. After implementation of a health education programme, the students’ knowledge of substance use has significantly increased and this confirms the success of the health education programme in improving students’ knowledge of substance use.

No significant association has been shown between the difference (gain) in knowledge scores and certain sociodemographic variables such as gender, age, and education of parents, occupational status of fathers, house ownership, and monthly family income. This result explains the possibility of applying the current education programme to all high school students, regardless of differences in sociodemographic variables.

Significant improvement in the students’ knowledge is consistent with what has been found in the previous studies. For example, Kavitha [22] reported that first and second-year students of Pre-University College in Indore demonstrated an increase in substance use knowledge after a health education programme. Similarly, Goswami et al [23] examined the effect of structured teaching programme on knowledge of nursing students regarding substance use and found that the intervention significantly improved students’ knowledge at one-week post-test. A further similar pattern of results was obtained in a study conducted by Isensee et al in Germany [24]. It has shown that a school-based prevention program improves adolescents’ smoking-related knowledge after the implementation of such a program. One more similar reported finding by Theou et al [25] also coincided with our findings, as students’ knowledge of substance use has obviously increased after implementation of an education program with a mean difference of 4.23. This result indicates the success of the current programme and this can be attributed to the content of the educational programme that was based on the previous literature relevant to the substance use and the methods used in the presentation of the programme, and most importantly the participation of the social workers “Rabars”, who played a vital role in educating students about the consequences of substance use [26].

This study has some limitations that should be considered. First limitation is the use of pre-test post-test design which is weak in evaluating the education intervention, in case of not using a control group. The second limitation of this study is an inability to follow the students and assess their knowledge over a long period of time, and that was because of the time of conducting the study (at the end of the academic year). In spite of these limitations, the present study provides a valuable contribution, because, to our knowledge, it is the first study examining an educational intervention to improve substance use knowledge of high school students in the Kurdistan Region of Iraq.

## Conclusions

Implementing a health education program about substance use on high school students in Erbil city had improved the knowledge of students about this topic. That is why a health education program about substance use is feasible to be implemented in high schools of Erbil. Thereby, proper implementation of this school-based prevention program is critical to strengthening protection and reducing the prevalence of substance use amongst this segment of the population.

## Ethical considerations

This study was conducted with approval from the research ethics committee at the college of medicine of Hawler medical university. A written permission was obtained from the Directorate of Education of Erbil to collect data. Prior to administering the surveys, written informed consent was obtained from the parents of all participant students. However, all students and their parents were assured that students’ participation is voluntary and the collected data are used only for the purpose of the present study, as well as for their benefit.

## Supporting information

S1 File Questionnaire (Kurdish and English). (PDF)
