# Supplementary material for: Impact of an education intervention on knowledge of high school students concerning substance use in Kurdistan Region-Iraq: A quasi-experimental study
Source: PLoS One. 2018 Oct 31;13(10):e0206063. doi: 10.1371/journal.pone.0206063 (PMC6209211; doi:10.1371/journal.pone.0206063)
Supplement: S1 File — (PDF) [file pone.0206063.s001.pdf]

کاریگەری پرۆگرامی هۆشیاری تەندروستی لە سەر ئاستی زانیاری قوتابیانی ئامادەیی لە شاری هەولێر سەبارەت بە ئاڵوودەبوون بە ماددە

تکایە دەست پێ بکە

١. **ژمارەی نهێنی قوتابی:** ....

٢. **ناوی قوتابخانە:** ....

❖ **هەندێك زانیاری دەربارەی خۆت**

٣. **رەگەز**

١. نێر ☐ ٢. مێ ☐

٤. **تەمەن** (   ) ساڵ

٥. **ئاستی خوێندن**

١. قوناغی چوارەم (١٠) ☐ ٢. قۆناغی پێنجەم (١١) ☐ ٣. قۆناغی شەشەم (١٢) ☐

پرسیارەکانی داهاتوو دەربارەی باوانەکانتە. ئەگەر تۆ بەزۆری لای باوانی چاودێری(زڕباب یان زڕدایك) پەروەردە کرایتەوە یان ئەوانی دیکە، وەلأم بۆ ئەوان بدەرەوە. بۆ نموونە، ئەگەر تۆ هەردووك زڕبابێك و باوکێکی سروشتیت هەیە، وەلأم بۆ کە یەك ئەوەی کە گرنگترین لە پەروەردە کردنەوەی بۆ تۆ.

٦. **ئاستی خوێندەواری باوك**

١. نەخوێندەوار ☐ ٤. ناوەندی ☐

٢. نوسین و خوێندنەوە ☐ ٥. ئامادەیی ☐

٣. بنەرەتی ☐ ٦. پەیمانگا/زانکۆ ☐

٧. **ئاستی خوێندەواری دایك**

١. نەخوێندەوار ☐ ٤. ناوەندی ☐

٢. نوسین و خوێندنەوە ☐ ٥. ئامادەیی ☐

٣. بنەرەتی ☐ ٦. پەیمانگا/زانکۆ ☐

٨. **پیشەی باوك**

١. بەکار ☐ تکایە جۆری کارەکەی بنوسە:............................

٢. بێکار ☐

٣. خانەنشین ☐ تکایە جۆری کاری پێشووی بنوسە:............................

٩. **موڵکداریەتی خانووی نیشتەجێبوون**

١. موڵك ☐ ٢. کرێ ☐

١٠. **ئۆتومبێلی خۆتان هەیە؟**

١. بەڵێ ☐ ٢. نەخێر ☐

١١. **داهاتی مانگانەی خێزان**

١. زۆر باش ☐ ٣. مام ناوەند ☐

٢. باش ☐ ٤. خراپ ☐

پرسیارەکانی خوارەوە دەربارەی زانیاریتە لە سەر دەرمان و ماددەهۆشبەرەکان، تکایە (یەك) وەڵامی ڕاست بۆ هەر پرسیارێك هەڵبژێرە:

١. **هەرکەسێك خوو بە هەرشتێك بگرێت بە ڕێژەیەکی زۆر و نەتوانێت وازی لێ بهێنێ، پێی دەڵێن:**

أ- ئالۆدەبوون ب- پاشەکشێ یان کشانەوە ج- ڕاهاتن یان قبوڵکردن د- شەرەنگێزی

٢. **بارێکی دەروونی و جەستەیی ناخۆش کە تووشی ئەو کەسانە دەبن کەدەست لەدەرمان و ماددە سڕکەرەکان هەڵدگرن و لەپڕ لێیان دەکشێنەوە، پێی دەڵێن:**

أ- ئالۆدەبوون ب- پاشەکشێ یان کشانەوە ج- ڕاهاتن یان قبوڵکردن د- شەرەنگێزی

٣. **گۆڕانکاریەکی فیسیۆلۆژی لە لەشی ئەو کەسەی کەدەرمان و ماددەسڕکەرەکان بەکاردەهێنێت، پێی دەڵێن:**

أ- ئالۆدەبوون ب- پاشەکشێ یان کشانەوە ج- ڕاهاتن یان قبوڵکردن د- شەرەنگێزی

٤. **ئەو دەرمانانەن کە پزیشك بە شێوەیەکی گشتی بۆ چارەسەرکردن بەکاریان دەهێنێت بەڵام خەڵك خراپ بەکاریان دەهێنن.**

أ- جگەرە ب- دەرمانە نۆژدارییەکان ج- دەرمانە سڕکەرەکان د- کهوول

٥. **لێکۆڵینەوەکان لەسەر هەڵسوکەوتی بەکارهێنەرانی دەرمان و ماددەسڕکەرەکان تێبینی کام لەم خاڵانەی لای خوارەوەیان کردووە:**

أ- شەرەنگێزی ب- ئاژاوەی ناوماڵ ج- قەرەباڵخبوونی گرتوخانەکان د- هەموو ئەوانە

٦. **بەپێی لێکۆلینەوەکان ئالۆودەبون هۆکاری زۆرە لەوانە:**

أ- کەمی ئاستی زانیاری ب- هامۆشۆی هاوڕێی خراپ ج- باوەڕ بە خۆ نەبوون د- هەموو ئەوانە

٧. **یەکێك لە جۆرەکانی دەرمان وماددەهۆشبەرەکان وریاکەرەوەکان Stimulants نمونەکانیان:**

أ- کۆکاین ب- هیرۆین ج- ئەلکهوول د- حەشیش

٨. **هێمنکەرەوەکان یان کپکەرەوەکان Depressants یەکێکن لە جۆرەکانی دەرمان وماددەهۆشبەرەکان نمونەکانیان:**

أ- کۆکاین ب- هیرۆین ج- ئەلکهوول د- حەشیش

٩. **مرۆڤ توشی دڵەڕاوکێ و وڕێنە دەکەن و هەستەکانی دەشێوێنن و شێوازی بینین و بیستن و هەستپێکردنی بۆ جیهانی دەوروبەری دەگۆڕن:**

أ- وریاکەرەوەکان ب- هێمنکەرەوەکان یان کپکەرەوەکان ج- هەلوسە هێنەرەکان د- هیچ کامێکیان

١٠. دەرمانێکی وریاکەرەوەیە بەڵام کاریگەریەکی هەلوسەیی سوکیشی هەیە

أ- ئێکستاسی ب- هیرۆین ج- ئەلکهوول د- کۆکاین

١١. لە گروپی وریاکەرەوەکان بە شێوەیەکی سروشتی بەکاردەهێنرێت، بەکارهێنانێکی زۆریان لەلایەن سەربازەکانەوە بۆ ئەوەی بەرەنگاری ئەو بێتاقەتی و پەستییە ببەنەوە کە بە "پەستینی شەڕ"ناسراوە. لەسەر شەقام و ناوبازاڕدا بەگەلێك ناو دەناسرێن وەك کریستال، خێرا؛

أ- ئێکستاسی ب- ئەمفیتانیەکان ج- کۆکاین د- حەشیش

١٢. لە گەڵای دەوەنێك دەردەهێنێت کە لە ناوە چە شاخاوییەکانی ئەمریکای باشوور لە وڵاتی وەکو پۆلیڤیا و کۆلۆمبیا و پیرۆ بەشێوەیەکی سروشتی دەڕوێت. پزیشکەکان بۆ سڕکردنی ناوچەیی (بەنجکردنی بەشێکی لەش) و دانسازی بەکاریان دەهێنا؛

أ- کۆکاین ب- هیرۆین ج- ئەلکهوول د- حەشیش

١٣. دەرمانێکە لە چا، قاوە، coca، گەلێك خواردنەوەی سوکی وەك کۆلا و گەلێك چوکلێتدا دەبینرێتەوە.

أ- هیرۆیین ب- کۆکاین ج- کۆدایین د- کافایین

١٤. جگەرە زیاتر لە ٤٠٠٠ ماددەی کیمیایی تێدایە، بە ماددەی ژەهراوی وەك ئەمۆنیاشەو، بەڵام تاوانباری سەرەکی لە جگەرەدا .... ئەم دەرمانە ئاڵودەکەرێکی بەهێزە

أ- قەتران ب- کاربۆن مۆنۆکساید ج- میسانۆل د- نیکۆتین

١٥. زۆربەی ئەو کەسانەی جگەرە دەکێشن تەمەنیان لە نێوان

أ- خوارووی ١٥ساڵ ب- ١٥ – ٢٥ ساڵ ج- ٢٦ – ٤٠ ساڵ د- سەرووی ٤٠ ساڵ

١٦. کام ڕۆژ لە هەموو ساڵێك دانراوە بۆ ڕۆژی بەرەگاربونەوەی جگەرە؟

أ- ٣١ حوزەیران ب- ٣١ ئایار ج- ٣١ مارس د- ٣١ ایلول

١٧. توێژینەوەکان دەریان خستوە کە .... بە شێوەیەکی تایبەت بۆ گەشەکردنی دڵ، سییەکان، مێشکی هەرزەکاران زیان بەخشە هەتا گەنجتر بی دەست دەدەیتە جگەرەکێشان ئەگەری توشبونت زیاترە بە نەخۆشییە کوژەرەکان.

أ- نیکۆتین ب- قەتران ج- کاربۆن دایۆکساید د- هەموو ئەوانە

١٨. ئامێرێکی جگەرەکێشانە کە توتنەکە بە هۆی خەڵوزەوە گەرم دەکرێت و دەسوتێنرێت ودووکەڵەکەی بەنێو بۆڕیەکەدا تێدەپەڕێت بەرەو نێو خانەیەکی پڕ لە ئاو

أ- Hookah ب- نێرگەلە ج- شیشە د- هەموو ئەوانە

١٩. لێکۆڵینەوە سەرەتاییەکان لەسەر نێرگەلە دەریان خستوە کە ئەو ئامێرە ڕادەی مەترسییەکانی ....بەرز دەکاتەوە و لێدانی دڵ خێراتر دەکات ئەگەری توشبوون بە نەخۆشیەکانی بۆڕییەکانی خوێن لە دڵدا زیاد دەکات و دەبێتە هۆی چەندەها جۆری شێرپەنجە، نابێت ئەوەمان بیر بچێت کە نێرگەلە تەنها هۆکارێکی دیکەی جگەرەکێشانە و هیچی تر.

أ- کاربۆن دایۆکساید و نیکۆتین ب- کاربۆن مۆنۆکساید و نیکۆتین ج- قەتران د- هیچ کامیان

٢٠. خواردنەوەی .... ئەگەری کارەسات زیاتر دەکات. بەتایبەتی وەك کارەساتی ڕێگاوبان کەوتنە خوارەوە، بەکارهێنانی ئامێری هەستیار و وردەکاری بەشێوەیەکی هەڵەو ترسناك.

أ- کۆکاین ب- هیرۆین ج- ئەلکهوول د- حەشیش

٢١. ئەو کەسەی ئاڵودەبووە بە کهوول پێی دەگوترێت؛

أ- مەستبوون ب- پاشەکشێ یان کشانەوە ج- ڕاهاتن یان قبولکردن د- ئەلکهوولیزم

٢٢. كام لە مانەی خوارەوە بە زیانەکانی ئلکهوول هەژماردەکرێت؛

أ- بەکارهێنانی توند و تیژی
ب- نەتوانینی بڕیاردان
ج- نەتوانینی کۆنترۆڵ کردن لەسەر ماسوولکەکانی لەش
د- هەموو ئەوانەی سەرەوە

٢٣. ئەو دەرمانانەن کە بە شێوەیەکی گشتی پزیشك دەیان نوسێت بۆ چارەسەری نەخۆشی وەك دڵەڕاوکێ و خەمۆکی و بێخەوی، پێیان دەگوترێت؛

أ- هێورکەرەوەکان ب- دەرمانە نوژدارییەکان ج- دەرمانە سڕکەرەکان د- هیچ کامیان

٢٤. ئەو مادانەن لە ڕێگەی لوتەوە هەڵدەمژرێن، هەندێکیان بناغەیەکی ئۆرگانیکی هەیە و لەکاتی هەڵمژیندا کاریگەرییەکی وەك ئەوەی ئەلکهوول یان ماددەی بەنجکەر دروست دەکەن؛

أ- هەڵمژینەکان و سکۆتینەکان ب- دەرمانە نوژدارییەکان ج- ئەلکهوول د- دەرمانە سڕکەرەکان

٢٥. یەکێکە لەگروپێکی دەرمان و ماددەسڕکەرەکان کە بە ئەفیونەکان opiates ناسراون. لە مۆرفین بەرهەمدێت و لە کاتێکدا کە پوختە. لە شێوەی تۆزێکی سپیدایە.

أ- کۆکاین ب- هیرۆین ج- ئەلکهوول د- حەشیش

٢٦. ڕوەکێکە کە لە گەلێك شوێنی ئەم جیهانە بە شێوەیەکی کێوی دەڕوێت. هەندێك کەس دەخاتە دڵەڕاوکێ و ترس و تۆقین و پاڕانۆیینەوە و وا هەست دەکەن کە هەموو کەس دژیانە.

أ- کۆکاین ب- هیرۆین ج- ئەلکهوول د- حەشیش

٢٧. دەرمانێکی سڕکەر یان بەنجکەری بەهێزە کە بۆ مەبەستی بەنجی نەشتەرگەری لە مرۆڤ و ئاژەڵ بەکارهاتوە. کاریگەری ئازارشکاندنی هەیە بەڵام کاردەکاتە سەر هەستەکانیش و بەو جۆرە کاریگەری هەڵوەسەهێنەریشی هەیە.

أ- پرۆکایین ب- هیرۆین ج- کێتەماین د- زایلۆکایین

٢٨. بەشێوەیەکی گشتی بەرەنگاربونەوەی دەرمان و ماددەسڕکەرەکان دەکرێت بەم شێوەیەی خوارەوە ڕێکبخرێت؛

أ- کۆنترۆڵ و چارەسەر
ب- کۆنترۆڵ، پەروەردە، چارەسەر
ج- پەروەردە وچارەسەر
د- کۆنترۆڵ و پەروەردە.

٢٩. بەردی بناغەی هەر پەروەردەیەکی پتەو لە .... وە دەس پێدەکات.

أ- قوتابخانە ب- خێزان ج- کۆمەڵگا د- هیچ کامیان

٣٠. کام لە مانەی خوارەوە دەتوان ڕۆڵی باش ببینن، و هەندێکیشیان بەردەوام بینیویانە، لە هۆشیارکردنەوەی کومەڵانی خەڵکدا و ڕێنمایی کردنی گەنجان بۆ بەدەستکەوتنی زانیاری ڕاست و دروست.

أ- تەلەفیزۆن و ڕادیۆ
ب- ئینتەرنێت
ج- گۆڤار و ڕۆژنامەکان
د- هەموو ئەوانەی سەرەوە

ENGLISH VERSION QUESTIONNAIRE

“Impact of an Education Intervention on Knowledge of High School Students Concerning Substance Use in Kurdistan Region-Iraq: A quasi-Experimental Study”

Please begin

The first questions ask for some BACKGROUND INFORMATION about yourself.

1. **Students’** serial number **……….…..**
2. **School name ……………….**
3. **Gender**
   1. Male ☐
   2. Female ☐
4. **Age in years** ………….. years
5. **Grade**
   1. Grade 10th ☐
   2. Grade 11th ☐

The next questions ask about your PARENTS. If you were raised mostly by foster parents, step-parents or others, answer for them. For example, if you have both a stepfather and a natural father, answer for the one who was the most important in raising you.

6. **Educational level of father**
   1. Illiterate ☐
   2. Read and write ☐
   3. primary school ☐
   4. Intermediate school ☐
   5. secondary school ☐
   6. Institute/College ☐
7. **Educational level of mother**
   1. Illiterate ☐
   2. Read and write ☐
   3. primary school ☐
   4. Intermediate school ☐
   5. secondary school ☐
   6. Institute/College ☐

8. Occupational status of father.

1. Employed ☐
2. Un employed ☐
3. Retired ☐

9. House ownership.

1. Owned ☐
2. Rented ☐

10. Monthly family income.

1. More than enough ☐
2. Enough ☐
3. Barely enough ☐
4. Not enough ☐

The following questions are about your knowledge of substance use. Please select one correct answer for each question

1- Anybody who is connected to something such that he/she cannot leave it is called:
a. Addiction
b. Withdrawal
c. Tolerance
d. Quarrel

2- A non-comfortable physical and psychological situation that happens to those who quit drugs, is called:
a. Addiction
b. Tolerance
c. Quarrel
d. Withdrawal

3- A physiological change that is happed to those who use drugs, is called:
a. Quarrel
b. Addiction
c. Withdrawal
d. Tolerance

4- The medicine that physicians prescribe for treatment but people use them improperly
a. Cigarette
b. Drug misuse
c. Anesthetic Drugs
d. Alcohol

5- What of the following points have been noticed by researches on drug users' behavior?
a. Quarrel
b. Troubles in the home
c. Crowded prisons
d. All the above

6- According to researches, addiction has many reasons including:
a. Low knowledge level
b. Bad friends
c. Non self-confidence
d. All the above

7- An example of the stimulants drug is:

a. Cocaine
b. Heroin
c. Alcohol
d. Cannabis

8- Depressants are considered types of drugs, for example:

a. Cocaine
b. Heroin
c. Alcohol
d. Cannabis

9- People get anxious, delirious; the way they feel, see, and listen change:

a. Stimulants
b. Depressants
c. Hallucinogens
d. None of the above

10- A stimulant medicine with a light hallucination

a. Ecstasy
b. Heroin
c. Alcohol
d. Cocaine

11- It is in the stimulants group and used naturally, mostly used by soldiers to face their tiredness and anger that is called "war anger". They are known by different names including Cristal:

a. Ecstasy
b. Amphetamine
c. Cocaine
d. Cannabis

12- Extracted from leaves of a plant that is naturally seen in the mountains of some of the states of South America such as Olivia, Colombia, and Peru State. Physicians and dentists use this material for local anesthesia:

a. Cocaine
b. Heroin
c. Alcohol
d. Cannabis

13- A medicine that is made from tea, coffee, cocoa, and other soft drinks like cola and is seen in chocolate:

a. Heroin
b. Cocaine
c. Codeine
d. Caffeine

14- Cigarette contains more 4000 chemical materials including poisonous material like ammonia, however, the main guilty person is …………… this medicine is a strong addictive.

a. Tar
b. Carbon monoxide
c. Methanol
d. Nicotine

15- Majority of those who smoke are aged between:

a. Below 15 years old
b. 15-25 years old
c. 40-26 years old
d. Above 40 years old

16- Which day is set as an anti-cigarette day?

a. June 13
b. May 31
c. March 31
d. September 31

17- Researches have shown that ……………. especially for heart, lung, and teenager' brain is harmful. The younger you start smoking, the more you are prone to fatal diseases

a. Nicotine
b. Tar
c. Carbon dioxide
d. All above**.**

18- A smoking tool by which the tobacco is used through coal and the smoke is passing through a pipe into canister full of water.

a. Hookah
b. Narghile

c. Shisha
d. All above.

19- Preliminary research results about hookah have stated that this tool lifts the risks of ………. and speeds up heart beats. Also, the diseases related to heart are increased. It causes several cancer diseases. Hence, the hookah is not more than another way of smoking

a. Carbon dioxide and nicotine
b. Carbon monoxide and nicotine
c. Tar
d. None of the above

20- Consuming ……………………….. increases the chances of accidents such as road accidents, falling, and improperly using sensitive devices.

a. Cocaine
b. Heroin
c. Alcohol
d. Cannabis

21- The person who is addicted to alcohol is called:

a. Drunk
b. Withdrawal
c. Getting used to or accepting it.
d. Alcoholism

22- Which of the following is considered disadvantages of alcohol?

a. Violence
b. Not being able to make decision
c. Unable to control muscle movement
d. All above

23- The medications that physician prescribe for diseases like anxiety, sadness, and insomnia are called:

a. Suppressant
b. Drug misuse
c. Anesthesia
d. None of the above

24- The substances that are inhaled by nose, some of them have organic base and during inhalation have impacts like drinking alcohol.

a. Inhalations and
b. Drug misuse
c. Alcohol
d. Anesthesia

25- One of the medications and drugs that is known as opiates. Produced from morphine as a white powder:

a. Cocaine
b. Heroin
c. Alcohol
d. Cannabis

26- A plant that is grown wildly. Makes some people anxious and freaky in such away they think that all people are against them:

a. Cocaine
b. Heroin
c. Alcohol
d. Cannabis

27- A drug or anesthetic substance used for surgery for human and animals. It is a pain killer but it affects the feelings:

a. Procaine
b. Heroin
c. Ketamine
d. Xylocaine

28- Generally, facing and combating drugs can be done as follows:

a. Control and treat
b. Control, educate, treat
c. Educate and treat
d. Control and educate

29- Foundation stone of any strong education starts from …………

a. School
b. Family
c. Society
d. None of the above

30- Which of the following can play a good role, some of them have already done, in educating people and guiding youth in getting proper information

a. Television and Radio
b. Internet
c. Journal and magazine
d. All above
